# Novel schizophrenia risk factor pathways regulate FEZ1 to advance oligodendroglia development

**DOI:** 10.1038/s41398-017-0028-z

**Published:** 2017-12-18

**Authors:** Xianjun Chen, Li Ku, Ruyi Mei, Guanglu Liu, Chongchong Xu, Zhexing Wen, Xiaofeng Zhao, Fei Wang, Lan Xiao, Yue Feng

**Affiliations:** 1 Department of Histology and Embryology, Chongqing Key Laboratory of Neurobiology, Third Military Medical University, Chongqing, 400038 China; 2 Department of Pharmacology, Emory University School of Medicine, Atlanta, GA 30322 USA; 3 Institute of Developmental and Regenerative Biology, Zhejiang Key Laboratory of Organ Development and Regeneration, College of Life and Environmental Sciences, Hangzhou Normal University, Hangzhou, 310036 China; 4 Department of Psychiatry and Cell Biology, Emory University School of Medicine, Atlanta, GA USA; 5 Department of Cell Biology, Emory University School of Medicine, Atlanta, GA USA

## Abstract

Neuropsychiatric disorders, represented by schizophrenia, affect not only neurons but also myelinating oligodendroglia (OL), both contribute to the complex etiology. Although numerous susceptibility genes for schizophrenia have been identified, their function has been primarily studied in neurons. Whether malfunction of risk genes underlies OL defects in schizophrenia pathogenesis remains poorly understood. In this study, we investigated the function and regulation of the well-recognized schizophrenia risk factor, Fasciculation and Elongation Protein Zeta-1 (FEZ1), in OL. We found that FEZ1 is expressed in oligodendroglia progenitor cells (OPCs) derived from rodent brains and human induced pluripotent stem cells (iPSCs) in culture and in myelinating oligodendrocytes in the brain. In addition, a vigorous upregulation of FEZ1 occurs during OPC differentiation and myelinogenesis, whereas knockdown of FEZ1 significantly attenuates the development of OL process arbors. We further showed that transcription of the Fez1 gene in OL cells is governed by a sophisticated functional interplay between histone acetylation-mediated chromatin modification and transcription factors that are dysregulated in schizophrenia. At the post-transcriptional level, the selective RNA-binding protein QKI, a glia-specific risk factor of schizophrenia, binds FEZ1 mRNA. Moreover, QKI deficiency results in a marked reduction of FEZ1 specifically in OL cells of the quakingviable (qk^v^) hypomyelination mutant mice. These observations have uncovered novel pathways that involve multifaceted genetic lesions and/or epigenetic dysregulations in schizophrenia, which converge on FEZ1 regulation and cause OL impairment in neuropsychiatric disorders.

## Introduction

Besides neuronal network defects, accumulating evidence from brain imaging and postmortem studies clearly demonstrates structural and functional impairment of myelinating oligodendroglia (OL) in major neuropsychiatric diseases^1^. Reduced OL density, aberrant expression of myelin-related genes, and white matter disruptions have been reported in the brains of schizophrenia, bipolar disease, and major depression patients^2–4^. Consequently, the contribution of OL and myelin defects on the complex etiology of psychiatric illnesses has been increasingly recognized in recent years. In fact, the first-episode schizophrenia patients already display compromised white matter integrity in cortical and subcortical brain regions^5–7^, suggesting that OL impairment is involved in early schizophrenia pathogenesis.

The identification of numerous schizophrenia-associated risk genes and familial transmission indicates the genetic component in schizophrenia pathogenesis^8,9^. However, the function of schizophrenia risk genes has been primarily investigated in neurons^10,11^. Whether malfunction of the same risk genes underlies OL impairment in schizophrenia pathogenesis remains elusive. In addition to the genetic variances, epigenetic abnormalities, including aberrant DNA and chromatin modifications, further increase the etiological complexity of psychiatric diseases^12^. Nonetheless, despite the key roles of epigenetic mechanisms in advancing OL development^13,14^, whether epigenetic dysregulation of schizophrenia risk factors affects OL function is not understood. These are important questions in understanding the OL-related and myelin-related pathogenesis of psychiatric diseases, but are unfortunately understudied.

The Fasciculation and Elongation Protein Zeta-1 (FEZ1) is a well-recognized schizophrenia risk factor. Genetic alterations in the FEZ1 gene are found in schizophrenia patients^15,16^. In addition, reduced FEZ1 expression is observed in schizophrenia postmortem brains^17^. The FEZ1 protein interacts with the disrupted in schizophrenia 1 (DISC1), which is indicated in multiple neuropsychiatric disorders, including schizophrenia^18^. In particular, an epistatic interaction between FEZ1 and DISC1 is reported to increase the risk of schizophrenia^19^. In animal models, genetic deletion of the mouse Fez1 gene results in hyperactivity and enhanced responsiveness to psychostimulants^20^. Functionally, FEZ1 belongs to a family of adhesion proteins known to govern neuronal axon growth and fasciculation^21^. Moreover, FEZ1–DISC1 interaction controls proper dendritic arbor growth of newborn neurons in the adult mouse hippocampus^19^. However, whether FEZ1 is expressed in OL cells and FEZ1 deficiency contributes to OL impairment in schizophrenia has not been investigated.

In this study, we report that FEZ1 is expressed in rodent and human OL lineage cells, which is vigorously upregulated during oligodendroglia progenitor cell (OPC) differentiation and myelinogenesis, essential for advancing OL development. Moreover, we identified a molecular orchestra that controls FEZ1 expression in OL by sophisticated transcriptional and post-transcriptional mechanisms, which contain multiple factors dysregulated in schizophrenia. These observations provide the first evidence indicating the functional importance of FEZ1 in OL. Furthermore, our study decodes the coordination of multiple schizophrenia-affected genes that converge on regulation of FEZ1 in OL, offering a model to explain how malfunction of distinct risk factors can lead to common abnormalities in the pathogenesis of psychiatric disorders.

## Materials and methods

### Animals

The quakingviable (qk^v^) mouse colony (Jackson Laboratory) and the transgenic mouse line that expresses the OL-specific Flag-QKI-6 transgene were described previously^22^. To introduce the transgene into the qk^v^/qk^v^ background (q/q), the male qk^v^/wt mice (q/w) that carry the QKI-6 transgene were mated with q/q female mice, and the q/q descendants that carry the QKI-6 transgene (q/qtg) were identified using PCR genotyping. Both male and female mice at the age of 2–3 months were used. All the animals within each genotype were randomly picked for experimental analysis. Animal treatment was according to the National Institutes of Health regulations under the approval of the Emory University Institutional Animal Care and Use Committee.

### Cell culture, treatment, and transfection

Immunopanning purification of OPCs was performed as previously described^23^. Primary cortical neurons were raised using embryonic day 18 Wistar rats^24^. The OPC cell line CG4 was propagated and induced for differentiation as previously described^25^. When indicated, primary cultured rat OPCs, neurons, and CG4 cells were treated with 50 ng/ml trichostatin A (TSA, Sigma) for 24 h before extraction of RNA or protein. PCDNA3.1, PC-Flag-SOX10, and PC-Flag-ID4 were transfected using Lipofectamine™ 2000 according to the manufacturer’s protocol (Thermo Fisher Scientific, Rockford, IL). Human induced pluripotent stem cell (iPSCs; passage ≤35) were cultured as previously described^26^. The detailed procedures for differentiation of human iPSCs into OPCs are described in Supplementary Information.

### Knockdown of FEZ1 by siRNA and lentivirus-expressed shRNA

A previously validated FEZ1 short interfering RNA (siRNA; 5′-GCTTGAGAATTTTTCTTCC-3′)^27^ or a negative control siRNA (ID# AM4611, Ambion) were co-transfected with EGFP-C2 (Clontech, CA) into CG4 cells. In addition, a lentivirus-encoded shRNA that targets the aforementioned FEZ1 mRNA sequence or a negative control shRNA was used to transduce rat primary OPCs. During acquisition, digital images were captured in randomly picked microscopic fields for each transfection experiment. Experimenters were blinded to treatment and analysis for cell morphology studies as described^25^. Days for various treatments of cultures are indicated in the corresponding legends.

### Immunoblot, immunocytochemistry, and RT-PCR

Immunoblot, immunocytochemistry PCR, and quantitative RT-PCR (RT-qPTR) were performed as previously described^28,29^. Antibodies and primers used are provided in the Supplementary Information.

### UV crosslinking immunoprecipitation and chromatin IP (ChIP)

Crosslinking immunoprecipitation (CLIP) was performed as previously described with modifications^22^. C57BL/6 mouse brainstems were minced and subjected to UV crosslinking. The post-nuclear supernatant was isolated from tissue lysates, precleared, and incubated with anti-Flag M2 beads (Sigma) for 2 h at 4 °C. After extensive washes, the immunoprecipitated complexes were eluted with 0.1 mg/ml Flag peptide. RNA was extracted and followed by RT-PCR.

For ChIP, cells were fixed in 3.4% formaldehyde in 1 × PBS buffer before being lysed and subjected to IP using anti-Histone H3-AcK9 antibody conjugated to protein A beads as described^24^. The immunoprecipitated genomic DNA was subjected to qPCR using primers specific to the Fez1 promoter region and was normalized to the amount of the input DNA of each sample.

### Identification of putative transcription factor-binding sites at the Fez1 promoter

JASPAR (http://jaspar.genereg.net/), a database of known transcription factor (TF)-binding sites from the experiment-based literature^30^, was used to predict putative TF binding to the rat Fez1 promoter (1000 bp upstream of the transcription start site; Supplementary Table S1). Relative profile score threshold was set as 80%, which is the default parameter in JASPAR database.

### Statistical analysis

All statistical analyses were performed with GraphPad Prism 5 software (GraphPad Software Inc.). For two-sample comparisons, independent two-sided *t*-test with or without Welch’s correction was applied. For three-sample comparisons, one-way ANOVA analysis and turkey post hoc test were used as indicated in the corresponding figure legends. All graphs were presented as means ± SEM. Statistical significances were indicated by *(*P* < 0.05) or **(*P* < 0.01).

## Results

### FEZ1 is expressed in OL lineage cells and deposited to the growing processes

**Fig. 1:**
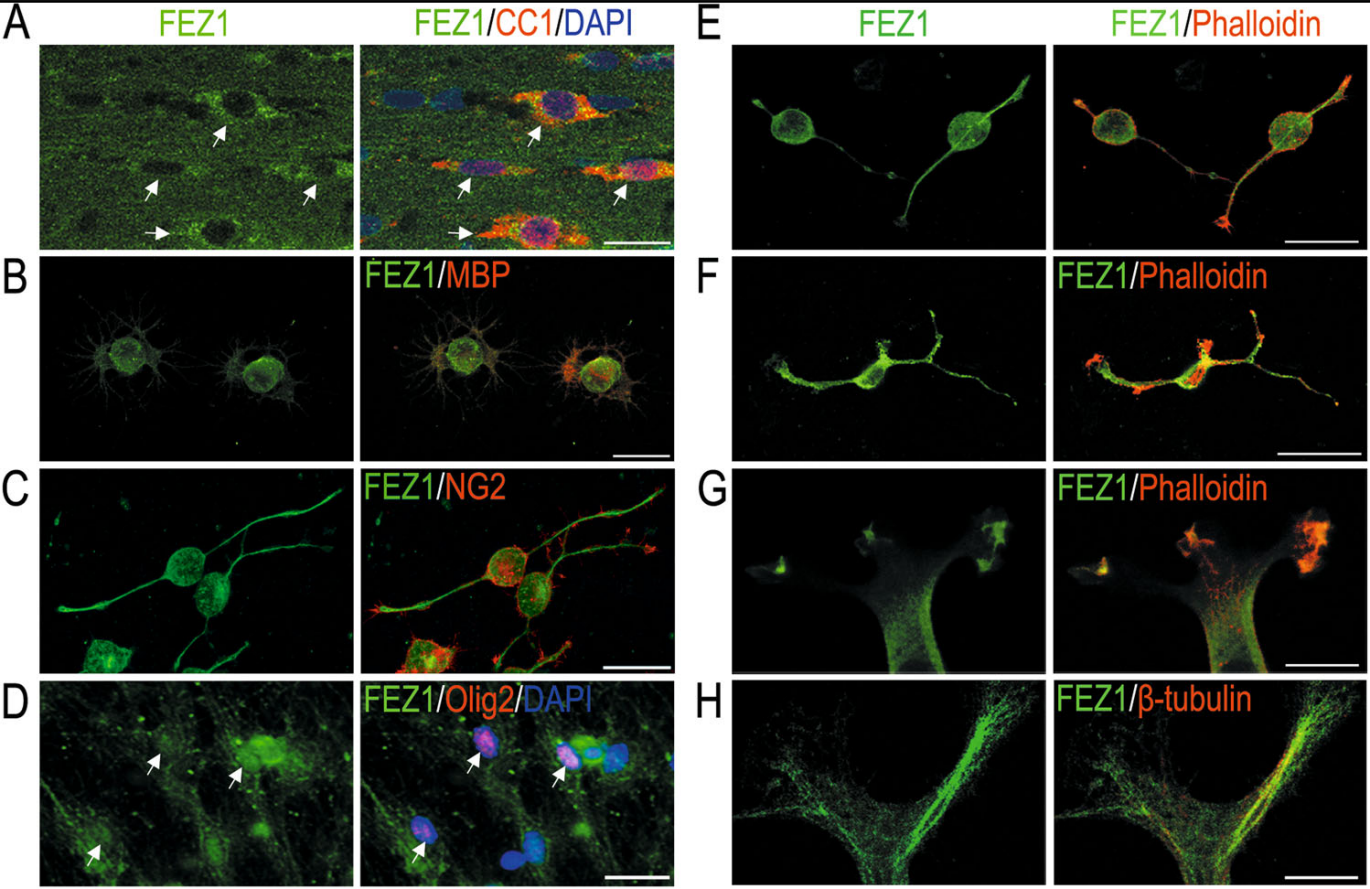
Expression and subcellular localization of FEZ1 in OL lineage cells. **a**–**d** Representative immunofluorescent images of FEZ1 (green) with OL markers (red) for different developmental stages. **a** Mature oligodendrocytes in the mouse corpus callosum marked by CC1; **b** primary cultured mature rat oligodendrocyte marked by MBP; **c** rat OPCs marked by NG2; and **d** human iPSC-derived OL marked by Olig2. **e**, **f** Enrichment of FEZ1 (green) in the enlarged distal tips of primary rat OPCs (**e**) and the OPC cell line CG4 (**f**). The leading edge is marked by phalloidin that stains F-actin (red). **g** A representative image for colocalization of FEZ1 (green) and phalloidin (red) in distal tips of CG4 processes. **h** Double-staining of FEZ1 (green) and β-tubulin (red) in CG4 processes. Scale bar in (**a**–**e**) 20 μm. Scale bar in F = 40 μm. Scale bar in (**g**, **h**) 10 μm

To determine whether FEZ1 is expressed in OL lineage cells, we first performed immunofluorescence (IF) staining using an antibody that recognizes the highly conserved FEZ1 protein (green) in rodents and human. OL cells at various differentiation stages were co-stained using well-established markers (red). FEZ1 was mainly detected in the cytoplasm of CC1-labeled mature OL cells in the mouse corpus callosum (Fig. 1a) and mature rat OL cells raised in primary culture that express myelin basic protein (MBP, Fig. 1b). Further analysis detected FEZ1 in rat OPCs (Figs. 1c, e), and OL differentiated from human iPSCs indicated by Olig2 staining (Fig. 1d). Notably, FEZ1 was deposited in the growing processes and enlarged tips (Figs. 1b, c, e). In addition, FEZ1 IF signals were also strongly detected in the growing processes of CG4 cells, a widely used OPC cell line that can be induced for morphogenetic differentiation to mimic primary OPCs (Fig. 1f). Colocalization of FEZ1 with F-actin in the tips of OPCs (Fig. 1e) and CG4 cells (Figs. 1f, g) was detected. Moreover, consistent with the microtubule-related function of FEZ1 in neurons^31^, FEZ1 colocalized with β-tubulin-labeled microtubule tracts in the distal processes of differentiating CG4 cells (Fig. 1h). These results demonstrate that FEZ1 is expressed in OL lineage cells and is associated with the cytoskeleton in OL distal processes.

### FEZ1 is upregulated during OL and myelin development, essential for advancing OL processes' development

**Fig. 2:**
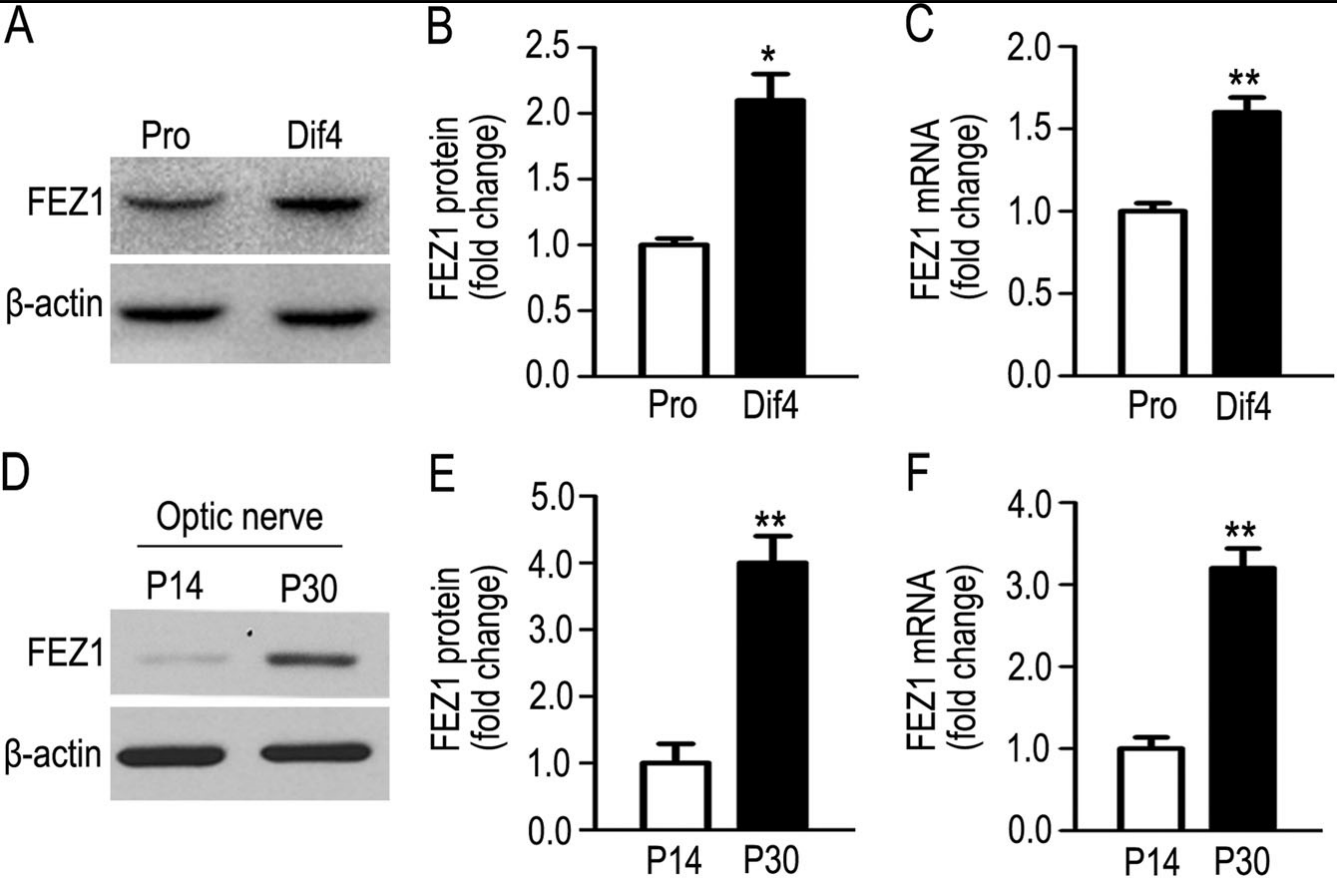
Upregulation of FEZ1 during OL differentiation in culture and myelin development in the optic nerve. **a** Representative immunoblot of FEZ1 protein in purified rat OPCs that underwent proliferation (Pro) or 4 days of differentiation in culture (Dif4). **b** Quantification of FEZ1 protein levels on immunoblot of primary OPC cultures (*n* = 4, Welch’s correction was applied). **c** Quantification of FEZ1 mRNA in primary OPC cultures by RT-qPCR (*n* = 4). **d** Representative immunoblot of FEZ1 protein in optic nerves on postnatal day 14 (P14) and day 30 (P30). **e** Quantification of FEZ1 protein levels on immunoblot of optic nerves (*n* = 4). **f** Quantification of FEZ1 mRNA in optic nerves by RT-qPCR (*n* = 5). β-actin was used as a loading control

To determine whether and how FEZ1 is regulated during OL development, we analyzed FEZ1 protein abundance by immunoblotting in primary cultured OPCs that underwent differentiation. As shown in Figs. 2a, b, FEZ1 protein levels were significantly increased on differentiation day 4 (Dif4) in comparison to undifferentiated proliferating OPCs (Pro). RT-qPCR further indicated increased FEZ1 mRNA on Dif4 (Fig. 2c). In addition, upregulation of FEZ1 protein was detected in optic nerves during the most vigorous myelinogenesis between postnatal days 14 and 30 (Figs. 2d, e), accompanied with similar folds of increase of FEZ1 mRNA (Fig. 2f). Although FEZ1 protein and mRNA were also upregulated during neuronal differentiation (Supplementary Fig. S1), FEZ1 mRNA in the optic nerve is primarily derived from the highly enriched oligodendroglial cells, whereas neuronal mRNAs are largely restricted in the soma and dendrites, but are negligible in mature axons. Thus, increased FEZ1 mRNA expression is likely the driving force for upregulation of FEZ1 protein in OL during differentiation and myelinogenesis.

**Fig. 3:**
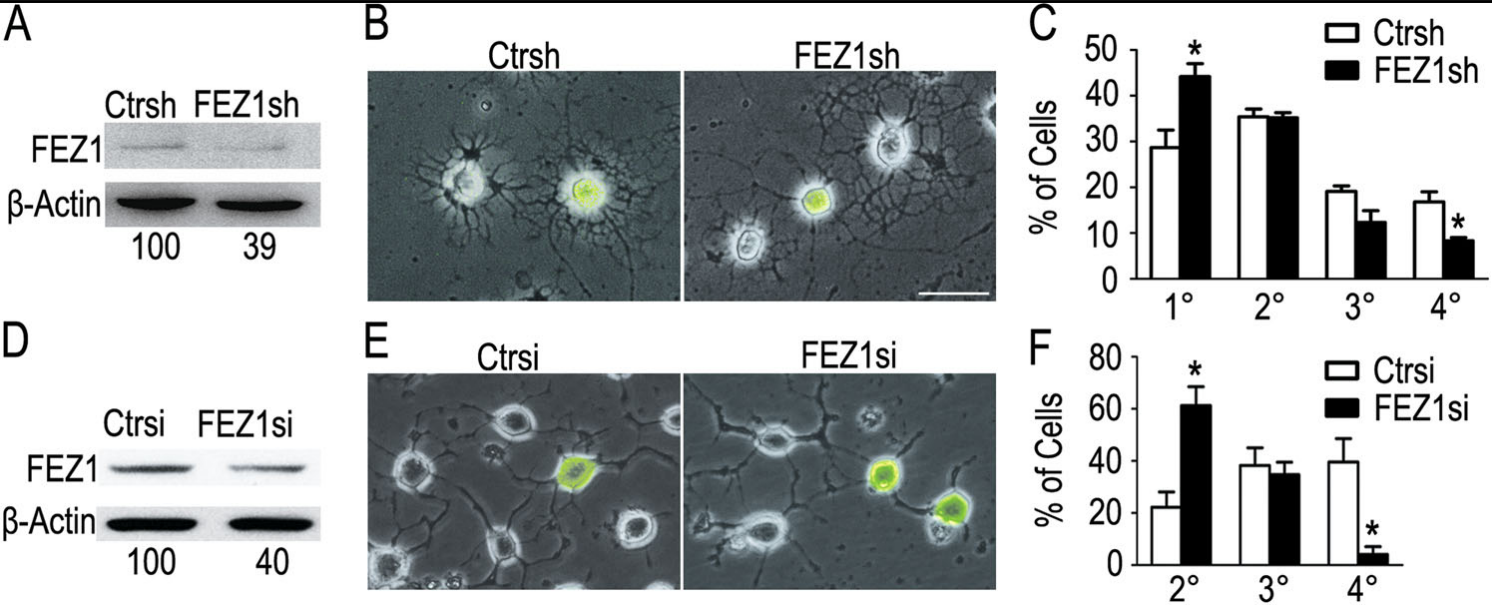
Knockdown of FEZ1 reduces the growth of OL processes. **a** Representative immunoblot of FEZ1 protein in primary OPCs treated by a lentivirus expressing control shRNA (Ctrsh) or FEZ1 shRNA (FEZ1sh). Densitometer readings of FEZ1 signal normalized to the loading reference β-actin are displayed underneath. **b** Representative images of primary OPCs treated with control shRNA or FEZ1 shRNA that underwent 72 h of differentiation. EGFP is expressed by the lentivirus for identification of infected cells. **c** Quantitative analysis of morphology of rat primary OPCs. Percentages of cells that harbor the highest order of process branches as primary (1°), secondary (2°), tertiary (3°), and quaternary (4°) were calculated. Data are expressed as mean ± SEM of three independent experiments (*n* = 353 cells in control shRNA, *n* = 390 cells in FEZ1 shRNA). **d** Representative immunoblot of FEZ1 protein in CG4 cells treated with control siRNA (Ctrsi) or FEZ1 siRNA (FEZ1si). Densitometer readings of FEZ1 signal normalized to the loading reference β-actin are displayed underneath. **e** Representative images of CG4 cells transfected with control siRNA or FEZ1 siRNA that underwent 48 h of differentiation. EGFP marks co-transfected cells. **f** Quantitative analysis of CG4 cell morphology as described in (**c**; *n* = 123 for each group). Scale bar = 20 μm

To directly access the function of FEZ1 in OL development, we generated a lentivirus that encodes a short-hairpin RNA (shRNA), specifically targeting a previously validated site in the FEZ1 mRNA^27^. Acute knockdown of FEZ1 (Fig. 3a) in purified rat OPCs significantly attenuated the growth and branching of OL processes (Figs. 3b, c). In addition, we also carried out siRNA-mediated knockdown of FEZ1 in CG4 cells (Fig. 3d), which can be induced for differentiation in a synchronized manner^32^. Similar to primary OPCs, knockdown of FEZ1 in CG4 cells significantly attenuated processes' complexity (Figs. 3e, f). However, the proliferation of OPCs was not affected by FEZ1 knockdown (Supplementary Fig. S2). Together, these results suggest that FEZ1 plays essential roles in promoting OL processes' arbor development during early differentiation.

### FEZ1 expression in OL is regulated by a functional interplay between histone deacetylation and TFs affected in psychiatric diseases

**Fig. 4:**
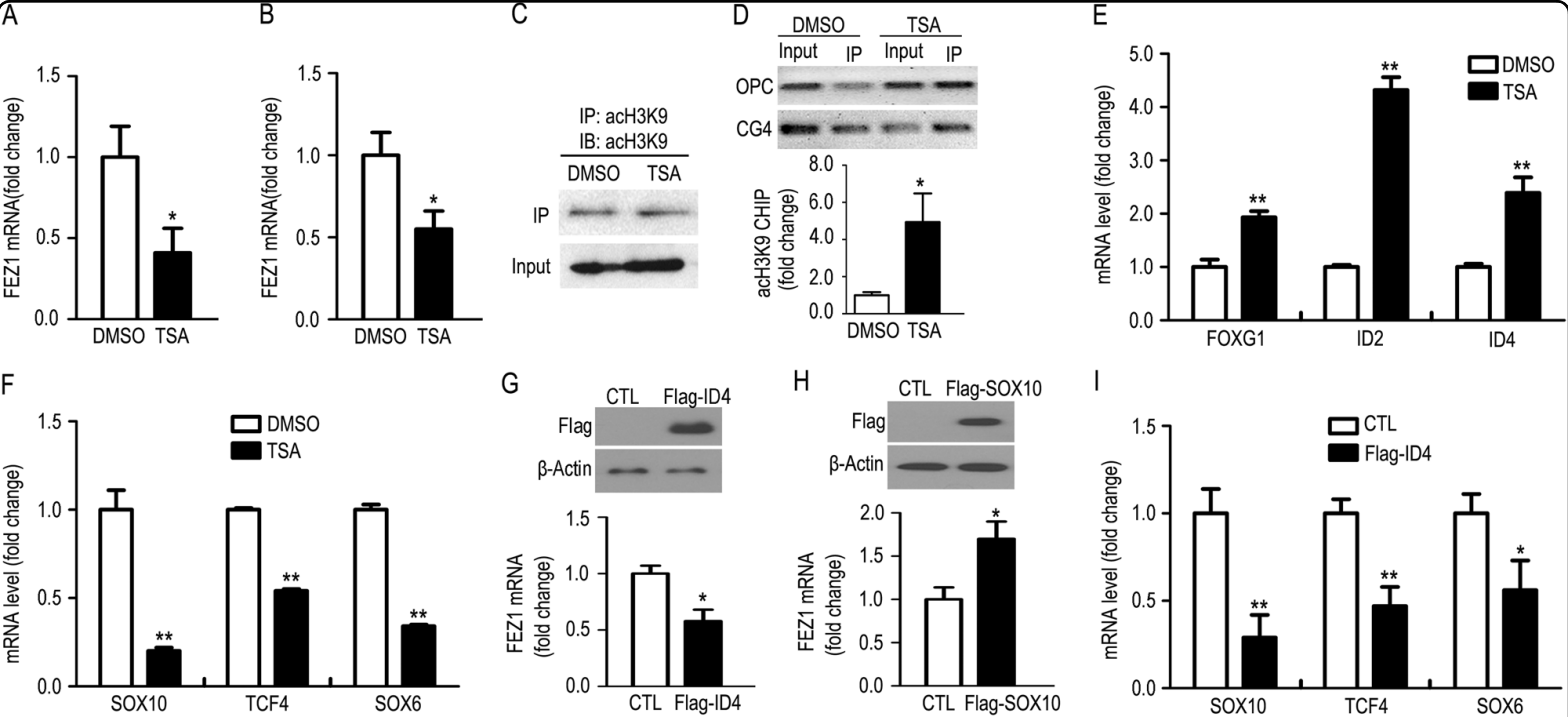
The cooperation between HDAC-dependent histone acetylation and transcription factors controls FEZ1 expression in OL. **a**, **b** FEZ1 mRNA levels in primary cultured OPCs that underwent 5 days of differentiation (DIV5; **a**; *n* = 4) and CG4 cells **b** (*n* = 5) were treated with TSA or DMSO for 24 h. **c** Western blot shows comparable efficiency of acH3K9 immunoprecipitation in DMSO or TSA-treated CG4 cells. **d** Representative illustration of increased acH3K9 at the FEZ1 promoter upon TSA treatment based on ChIP–PCR (upper panel) in rat OL and CG4 cells. ChIP–qPCR quantification of acH3K9 at the FEZ1 promoter in CG4 cells was normalized to the total input of genomic DNA and was graphically displayed (lower panel, *n* = 7). **e**, **f** RT-qPCR quantification of transcription repressor (FOXG1, ID2, ID4) mRNAs **e** and transcription activator (SOX10, TCF4, SOX6) mRNAs **f** in DMSO-treated or TSA-treated CG4 cells (*n* = 5, Welch’s correction was applied). **g** Representative immunoblot of Flag-ID4 expressed in CG4 cells (upper panel), which leads to downregulation of the FEZ1 mRNA detected by RT-qPCR (lower panel, *n* = 6 in CTL, *n* = 8 in Flag-ID4). **h** Representative immunoblot of Flag-SOX10 in CG4 cells (upper panel), which increases FEZ1 mRNA detected by RT-qPCR (lower panel, *n* = 5). **i** Expression of Flag-ID4 in CG4 cells leads to downregulation of the activator TFs (SOX10, TCF4, SOX6) mRNAs detected by RT-qPCR (*n* = 8 in CTL, *n* = 6 in Flag-ID4)

Considering the critical roles of histone deacetylase (HDAC)-dependent transcriptional regulation in advancing OL differentiation, we next questioned whether FEZ1 is regulated by HDAC activity in OL. Differentiating rat OL cells were treated with the HDAC inhibitor TSA. Surprisingly, in contrast to the well-documented activity of TSA for transcriptional induction through histone acetylation-mediated chromatin modification^33^, a significant downregulation of FEZ1 mRNA was observed in TSA-treated primary OL cells (Fig. 4a) and CG4 cells (Fig. 4b). Immunoblot confirmed an overall increase of acetylated histone 3 at K9 (acH3K9) by TSA (Supplementary Fig. S3A). In contrast, TSA treatment did not change FEZ1 mRNA levels in primary cultured cortical neurons (Supplementary Fig. S4), suggesting that HDAC-dependent regulation of FEZ1 is OL-specific. Conversely, TSA markedly induced the mRNAs of the cyclin-dependent kinase 5 activator P39 in neurons (Supplementary Fig. S3C). In addition, chromatin immunoprecipitation (ChIP)-qPCR of acH3K9, a surrogate marker for increased accessibility of genomic DNA to TFs^34^, demonstrated effective immunoprecipitation of overall acH3K9 (Fig. 4c) but increased acH3K9 at the Fez1 promoter region in OPCs and CG4 cells (Fig. 4d). These results indicate that TSA-induced increase of FEZ1 promoter accessibility leads to OL-specific FEZ1 downregulation; raising an intriguing possibility that FEZ1 transcription may be predominantly regulated by transcription repressors in OL cells.

We searched for TFs that may bind rat Fez1 promoter, especially focusing on repressor TFs that decline during OPC differentiation and thus may underlie the developmentally programmed FEZ1 upregulation. The JASPAR algorithm identified 349 TFs predicted to bind both the rat and the human Fez1 promoter, respectively (Supplementary Fig. S5A). Approximately 90% of these TFs are predicted to bind a 500 bp region immediately upstream of the Fez1 transcription start site, which is highly conserved in rat and human (Supplementary Fig. S5B). We found that 106 of the predicted Fez1 promoter-binding TFs were expressed in OPC and mature OL cells documented by RNA-seq (Supplementary Fig. S5C)^35^. A majority of these OPC-expressed Fez1 promoter-binding TFs were downregulated in mature OL. In addition, 30 predicted Fez1 promoter-binding TFs were found in an independent microarray study that documents transcriptome changes at specific stages of OPC differentiation (Supplementary Fig. S5D and Supplementary Table S1)^36^. When cross-referenced, 20 predicted Fez1 promoter-binding TFs were found in both OL databases, which are regulated during early OL differentiation along with FEZ1 upregulation. Strikingly, a majority of these predicted Fez1 promoter-binding TFs were dysregulated in neuropsychiatric disorders (Supplementary Table S1).

We selected a number of developmentally regulated Fez1 promoter-binding TFs identified in both OL databases and dysregulated in neuropsychiatric diseases, and tested whether they are regulated by TSA thus may underlie FEZ1 downregulation. Interestingly, TSA increased expression of several repressor TFs that are predicted to bind Fez1 promoter, including FOXG1, ID2, and ID4 (Fig. 4e). Conversely, activator TFs predicted to bind Fez1 promoter were downregulated by TSA, including SOX10, TCF4, and SOX6 (Fig. 4f). Notably, previous studies reported an aberrant increase of the repressor TF ID4 in schizophrenia patient brains and reduction of the OL-specific activator TF SOX10 in the brains of schizophrenia, bipolar, and major depression postmortem cohorts (Supplementary Table S1). We thus tested whether ID4 and SOX10 can regulate endogenous FEZ1 expression. Indeed, Flag-ID4 reduced FEZ1 mRNA in CG4 cells (Fig. 4g), whereas Flag-SOX10 increased FEZ1 mRNA (Fig. 4h). Moreover, consistent with the idea that repressor TFs play dominant roles, Flag-ID4 significantly downregulated multiple OL-specific activator TFs that are predicted to bind FEZ1 promoter (Fig. 4i). Thus, ID4-mediated downregulation of FEZ1 may involve direct transcriptional inhibition of FEZ1 by binding the FEZ1 promotor, as well as a cross-suppression of FEZ1 by reducing activator TFs that function to enhance FEZ1 transcription. Together, these data suggest that FEZ1 transcription in OL is governed by functional cooperation between HDAC-dependent chromatin modulation and OL-specific TFs that are susceptible to dysregulation in neuropsychiatric diseases.

### The Quaking I (QKI) RNA-binding protein is essential for FEZ1 expression in OL

**Fig. 5:**
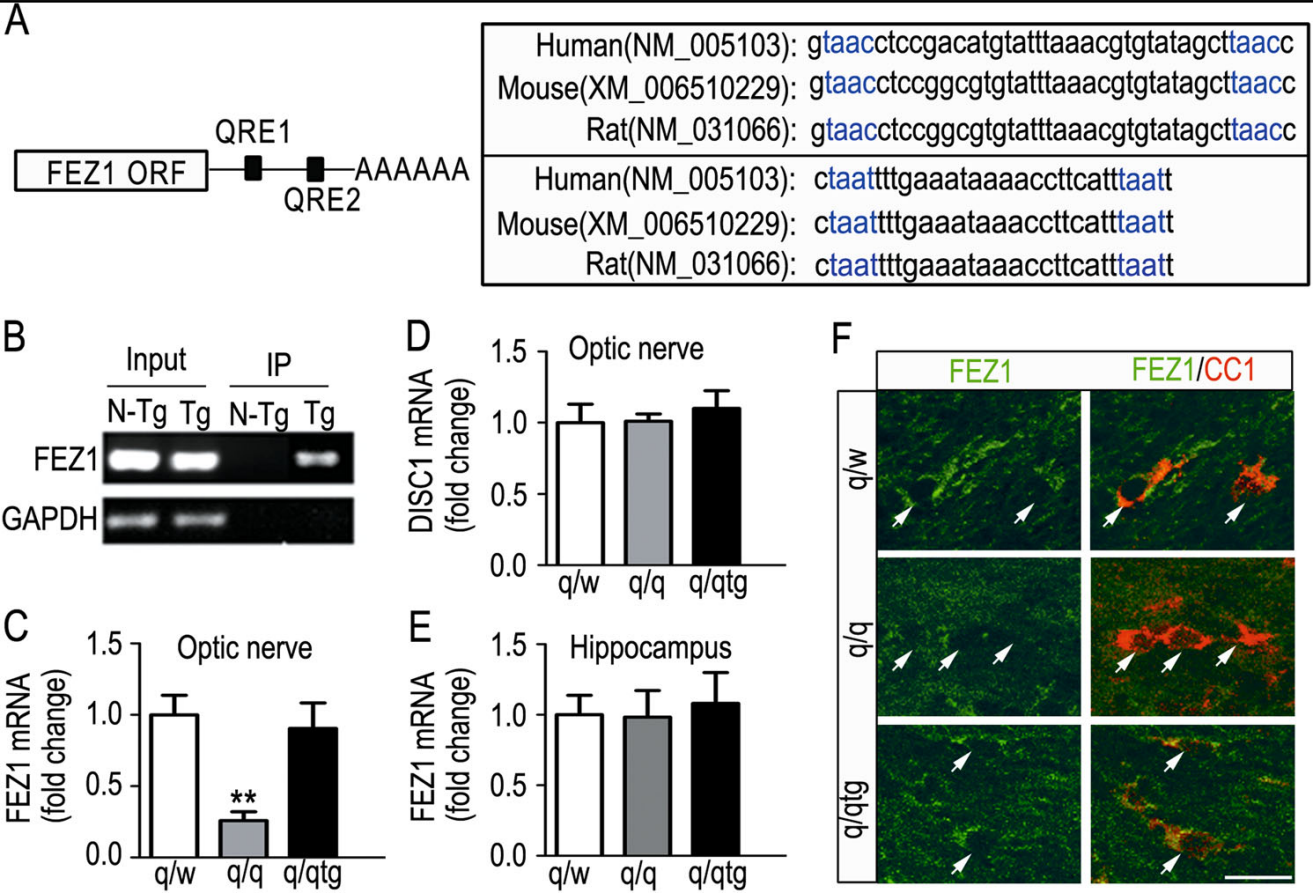
Post-transcriptional regulation of FEZ1 mRNA by cytoplasmic QKI. **a** Schematic illustration (left) of two Quaking response elements (QRE) located at 3’ UTR of FEZ1 mRNA. The QRE motif sequences of human, mouse, and rat QRE motifs are aligned on the right. The consensus QRE sequences are marked in blue. **b** UV-crosslink immunoprecipitation (CLIP) followed by RT-PCR revealed that interaction of FEZ1 mRNA with Flag-QKI-6 expressed specifically in brain OL of transgenic mice (Tg). Non-transgene (N-Tg) mice were used as a negative control. RT-PCR of the GAPDH mRNA that does not interact with QKI-6 was used as a negative control for specificity. **c** QKI deficiency in the homozygous quakingviable (qk^v^) mutant mice (q/q) affects FEZ1 mRNA abundance in OL, which can be rescued by the Flag-QKI-6 transgene. The FEZ1 mRNA levels in optic nerves of qk^v^/WT (q/w), qk^v^/qk^v^ (q/q), and q/qtg mice (*n* = 6) were quantified by RT-qPCR followed by one-way ANOVA analysis and Tukey post hoc test. **d** RT-qPCR of DISC1 mRNA in the optic nerves of q/w, q/q, and q/qtg mice (*n* = 4). **e** RT-qPCR of FEZ1 mRNA in the hippocampus of q/w, q/q, and q/qtg mice (*n* = 4). **f** Immunocytochemistry of FEZ1 (green) in mature oligodendrocytes marked by CC1 (red) in the corpus callosum of q/w, q/q, and q/qtg mice. Arrows indicated diminished FEZ1 protein in CC1 + cells from the q/q mice. Scale bar = 25 μm

Besides transcription, post-transcriptional mechanisms also play key roles in controlling mRNA abundance and promoting OL development. We found that human, mouse, and rat FEZ1 3’ untranslated regions harbor two consensus Quaking response elements (Fig. 5a). These are well-known sequence motifs controlling mRNA stability through interaction with the RNA-binding protein QKI, a glia-expressed schizophrenia risk factor^37,38^, essential for OL and myelin development^25^. Indeed, FEZ1 mRNA was co-immunoprecipitated with the Flag-tagged QKI-6 cytoplasmic isoform (Tg, Fig. 5b), which is specifically expressed in OL^22^. Consistent with the essential role of QKI in stabilization of its mRNA ligands, FEZ1 mRNA was markedly reduced in optic nerves of the homozygous quakingviable (qk^v^) hypomyelination mutant mice (q/q, Fig. 5c) that harbor OL-specific QKI deficiency^39^. The reduction of FEZ1 in q/q optic nerves was completely rescued by Flag-QKI-6 (q/qtg, Fig. 5c)^22^. Unlike FEZ1, DISC1 mRNA was not regulated by QKI (Fig. 5d). Moreover, in contrast to optic nerves, FEZ1 expression was not affected by QKI deficiency in the hippocampi of the q/q mutant mice (Fig. 5e), which harbor small numbers of OL cells but enriched in neurons. Hence, QKI deficiency only affected FEZ1 expression specifically in OL but not in neurons. Furthermore, IF staining demonstrated diminished FEZ1 protein in CC1 + mature OLs in the corpus callosum of the q/q mutant mice, which can be rescued by transgenic expression of Flag-QKI-6 (Fig. 5f). These results suggest that QKI binds and stabilizes FEZ1 mRNA in the OL cytoplasm, hence connecting the function of FEZ1 in OL with this glia-specific schizophrenia risk factor at the level of post-transcriptional regulation.

## Discussion

Our studies revealed that FEZ1 is expressed in the OL lineage throughout development and plays essential roles to promote formation of OL process arbors. Moreover, we identified a number of sophisticated molecular pathways that cooperate to govern FEZ1 expression in OL at transcriptional and post-transcriptional steps, linking FEZ1 with multiple regulators crucial for governing OL development but susceptible to genetic mutations and/or epigenetic dysregulation in psychiatric disorders. A working model for this multifactorial susceptibility mechanism of psychiatric illnesses is illustrated in Supplementary Figure S6.

Although the function of FEZ1 in neuronal development is well documented^19,21^, previous studies did not address whether FEZ1 is expressed in OL and how FEZ1 deficiency may be connected with OL impairment in neuropsychiatric diseases. Here we showed for the first time that FEZ1 is expressed in rodent and human OL lineage cells. In addition, acute knockdown of FEZ1 in OPCs significantly attenuated development of OL processes, suggesting an essential role of FEZ1 in early OL differentiation. Given the highly conserved FEZ1 amino-acid sequence (data not shown), FEZ1 most likely also functions to promote human OL development. In this regard, genetic alterations that result in FEZ1 deficiency and/or malfunction will affect not only neurons but also OL lineage cells, which contribute to the OL etiology in psychiatric diseases.

The roles of FEZ1 in promoting the growth of OL processes recapitulate its well-characterized function in axonal and dendritic arbor formation of neurons^19,21,40^, suggesting a functional analogy of FEZ1 in both the neuronal and the OL lineages. The marked upregulation of FEZ1 during differentiation of both OL and neurons suggests an increased functional requirement of FEZ1 to support the most vigorous growth of neural cell processes. In the developing hippocampal neurons, FEZ1 is found in growth cones and is associated with F-actins^41^. More recent studies revealed that FEZ1 also acts as a kinesin-1 adaptor and plays important roles in axonal transport and presynaptic specification^31,42^. We found that FEZ1 colocalized with F-actin and microtubule fibers in the growing processes and distal tips of OL cells, suggesting that FEZ1–cytoskeleton interaction likely underlies OL process development, similar to its function in neuronal network formation.

Establishing the complex OL process arbors is an important step before myelin sheath formation^43^. Myelinogenesis in vivo requires coordination between oligodendrocytes and axons, which involves both cell autonomous mechanisms and OL–axon interactions^44^. We observed a marked increase of FEZ1 during differentiation of purified neurons and OL in culture. Moreover, FEZ1 is upregulated in the optic nerve during rigorous myelination (Fig. 2), suggesting that FEZ1 may also promote myelin formation. Thus, FEZ1 upregulation may serve as a mechanism for coordination between myelinating oligodendrocytes and neurons during myelinogenesis. Whether FEZ1 indeed promotes myelin development in vivo still remains unexplored. Conventional knockout of Fez1 in mice causes schizophrenia-related behavioral abnormalities without gross defects in the adult brain^20^. However, Fez1 function in OL and myelin development in vivo has not been carefully examined, which warrants future investigation in a conditional Fez1 knockout mouse model. It is important to point out that, besides the classical function of myelination, the non-myelinating roles of OL also modulate neuronal function, such as trophic and/or metabolic support and neurotransmitter release^45–48^. Whether FEZ1 may also support non-myelination functions of OL is the next challenge for future studies.

FEZ1 expression in OL is regulated by a sophisticated molecular orchestra. First, HDAC inhibition increases histone acetylation at the FEZ1 promoter but reduces FEZ1 mRNA specifically in OL cells. The TSA-induced FEZ1 downregulation recapitulates OL genes that play key roles in promoting OL differentiation, such as Sox10 and Tcf4 (Fig. 4). In contrast, HDAC inhibition selectively enhances expression of repressor genes represented by Id4, which were downregulated during early OL differentiation. Hence, in contrast to TSA-induced neuronal differentiation^49^, TSA ablates OL differentiation and myelinogenesis^50^. It is postulated that transcription in OPCs is predominantly controlled by repressor TFs that prevent OL differentiation. HDAC-mediated histone deacetylation causes chromatin condensation in OPCs, which downregulates differentiation suppressor TFs and reduces their accessibility to differentiation-promoting genes, including FEZ1, to permit OL differentiation^51^. Besides direct suppression of FEZ1, repressor TFs also cross-inhibit OL-specific activator TFs that act to increase FEZ1 transcription (Fig. 4). The conserved Fez1 promoter sequence and TFs in rat and human suggest that the rat OL transcriptional pathways that regulate FEZ1 identified here are also present in human OL. Interestingly, a number of repressor TFs predicted to bind Fez1 promoter are aberrantly increased in brains of mental illness, whereas activators TFs for FEZ1 are reduced (Supplementary Table S1), similar to the effects in OL upon TSA treatment. This raises an intriguing possibility that aberrant histone acetylation, perhaps due to malfunction of HDACs, may selectively affect multiple key players in OL development beyond FEZ1, which underlies OL-specific etiology of mental illnesses.

Besides connecting FEZ1 with TFs that are dysregulated in psychiatric disorders, our studies also identified FEZ1 as a downstream target of QKI-dependent post-transcriptional regulation, which is essential for OL differentiation and myelinogenesis^25,52^. QKI is a glia-specific risk factor for schizophrenia, not expressed in brain neurons^53^. In addition to the clusters of single-nucleotide polymorphisms in human QKI that co-segregate with schizophrenia, reduced QKI expression is observed in the postnatal brains of multiple schizophrenia cohorts^37,54^. There are three QKI isoforms derived from alternative splicing^39^, which display distinct nuclear–cytoplasmic distribution and regulate target mRNAs at various post-transcriptional steps^22^. The human and mouse QKI protein isoforms are identical. The q/q mutant mice harbor deficiency of all QKI isoforms and diminished FEZ1 mRNA specifically in OL cells, which can be rescued by the cytoplasmic isoform QKI-6, suggesting that QKI controls FEZ1 mRNA stability in the OL cytoplasm. Together, our results reveal an OL-specific mechanism for regulating FEZ1 expression at transcriptional and post-transcriptional levels; both are susceptible to dysregulation in schizophrenia.

A rapidly growing list of susceptibility genes for schizophrenia has been identified. How malfunction of distinct risk factors leads to the common pathophysiology in schizophrenia is a prevailing question in understanding the pathogenesis of psychiatric disorders. The sophisticated regulatory pathways that secure the abundance of FEZ1 in OL contain numerous risk factors affected in psychiatric diseases. Thus, malfunction of different factor(s) can converge on FEZ1 deficiency in schizophrenia. In this regard, our study on FEZ1 provides an example how multiple risk factors are functionally connected in schizophrenia, which leads to OL impairment.

## Electronic supplementary material

Spplemental material for 2017TP000313, Chen et al.
