# Supplementary material for: Novel schizophrenia risk factor pathways regulate FEZ1 to advance oligodendroglia development
Source: Transl Psychiatry. 2017 Dec 18;7:1293. doi: 10.1038/s41398-017-0028-z (PMC5802537; doi:10.1038/s41398-017-0028-z)
Supplement: Supplementary file 1 — Spplemental material for 2017TP000313, Chen et al. [file 41398_2017_28_MOESM1_ESM.doc]

**SUPPLEMENTARY FIGURES, TABLES AND LEGENDS**

**Supplementary Figure S1.** FEZ1 expression during neuronal development. (A) Representative immunoblot of FEZ1 protein in mouse hippocampi at postnatal day5, 10 and 30 (upper panel). FEZ1 protein levels on immunoblots are quantified (lower panel, n=4 in P5, n=3 in P10 or P30). (B) RT-qPCR of FEZ1 mRNA in mouse hippocampi at the aforementioned time points (n=4 in P5, n=3 in P10 or P30). One-way ANOVA analysis and Tukey post-hoc test were used in both A and B. (C) Representative immunoblot of FEZ1 protein in primary cultured rat cortical neurons at differentiation day5 (DIV5) and 15 (DIV15) (upper panel). FEZ1 protein levels on immunoblots are quantified (lower panel, n=4). (D) RT-qPCR quantification of FEZ1 mRNA in primary cultured rat cortical neurons (n=3). For all quantification, β-actin is used as a loading reference. Data are expressed as mean ± s.e.m. *p<0.05; **p<0.01.

**Supplementary Figure S2.** Knockdown of FEZ1 did not affect OPC proliferation. (A) Representative immunofluorescent images of OPCs after infection of lentiviruses that express EGFP and control shRNA (Ctrsh) or FEZ1 shRNA (FEZ1sh) for 48 hours. Cells were immunostained for Ki67 (red), Olig2 (white) and DAPI (blue). Scale bar=50μm. (B) Percentage of Ki67+ proliferating OPCs in all infected OLs were calculated from three independent experiments (n=281 cells for Ctrsh, n=363 cells for FEZ1sh). Data are expressed as mean ± s.e.m. ns, no significant difference.

**Supplementary Figure S3.** TSA-treatment increases overall histone acetylation in CG4 cells. (A) Immunoblot detects acetylated H3K9 at lysine 9 (acH3K9) in CG4 cells after exposure to DMSO- or TSA-treatment for 24 hrs. (B) The levels of acH3K9 were normalized to the housekeeping protein translation initiation factor 5α (eIF5α) and quantitatively displayed (n=6). Data are expressed as mean ± s.e.m. **p<0.01.

**Supplementary Figure S4.** TSA-treatment does not alter FEZ1 mRNA expression in neurons. Primary cultured rat cortical neurons at differentiation day5 (DIV5) were treated with TSA or DMSO for 24 hours. RT-qPCR quantification of mRNA levels of FEZ1 (A), brain derived neurotrophic factor (BDNF) (B), and P39 (C) were performed (n=6). Data are expressed as mean ± s.e.m. **p<0.01.


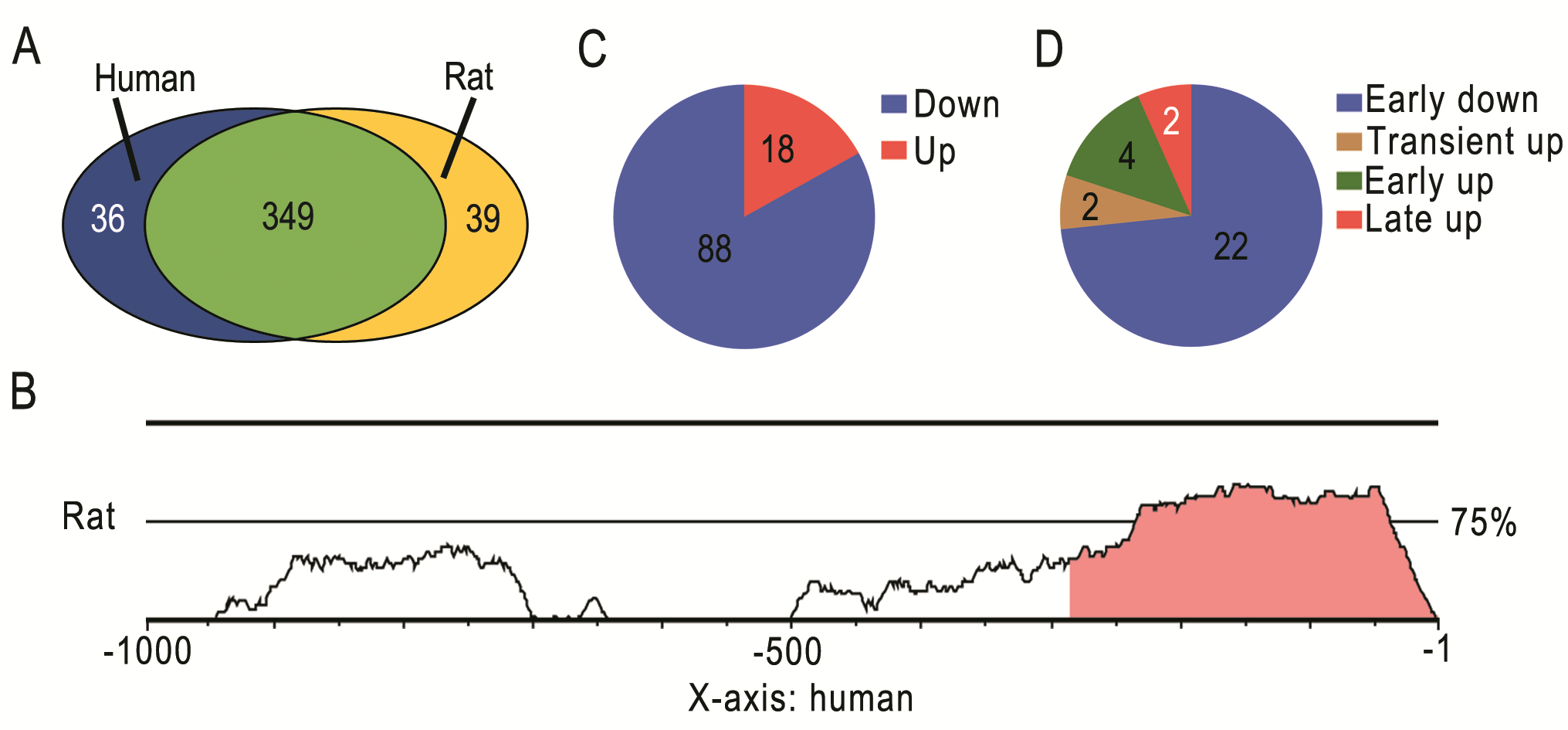


**Supplementary Figure S5.** (A) Venn diagram shows TFs predicted for binding rat and human FEZ1 promoter. (B) Vista plot indicates sequence homology between rat and human FEZ1 promoter region. (C) Percent distribution of up-regulated (red) and downregulated (blue) TFs in MOG+ mature oligodendrocytes as compared with OPCs.[^1^](#_ENREF_1) (D) Percent distribution of TFs that are regulated at specifically defined developmental stages in primary cultured OL from day0 to day9.[^2^](#_ENREF_2) The exact number of TFs was shown in each part of the corresponding pie charts.


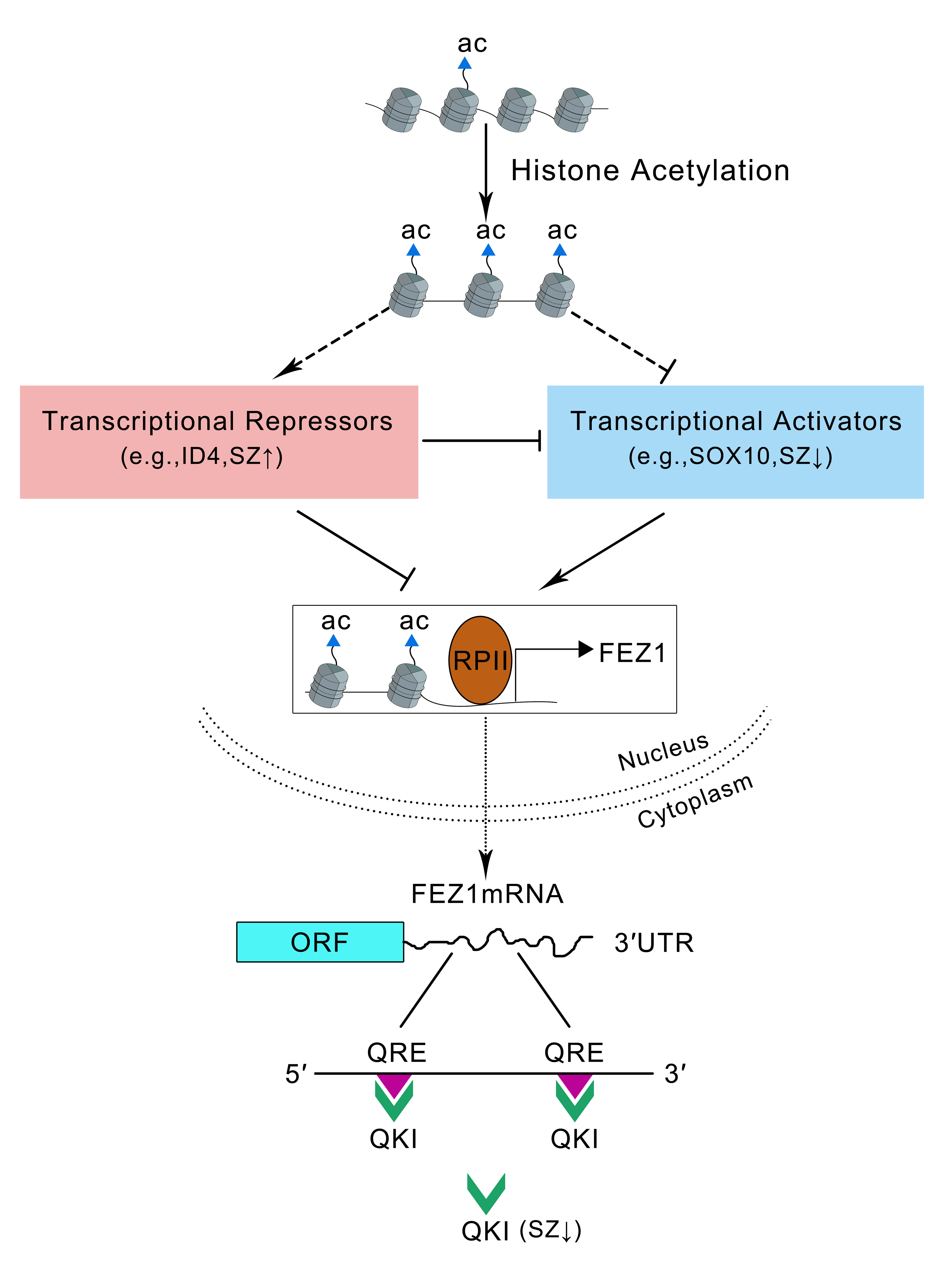


**Supplementary Figure S6.** A working model for FEZ1 regulation by OL-specific molecular pathways affected in schizophrenia. Histone acetylation upregulates transcription repressors but downregulates transcription activators that bind the Fez1 promoter. Transcription repressors (e.g. ID4) play predominant roles in regulating FEZ1 through direct binding to the Fez1 gene promoter and indirect suppression of transcription activators (e.g. SOX10) that act on the Fez1 promoter. In addition, the glia-specific RNA-binding protein QKI stabilizes the FEZ1 mRNA in OL cytoplasm. Such a multifaceted molecular orchestra governs normal FEZ1 expression in OL lineage cells and includes many regulatory factors affected in schizophrenia thus contribute to OL impairment. ac, acetyl group; ORF, open reading frame; UTR, untranslated region; QRE, quaking response element.

**Supplemental Table S1.** Dynamic regulation of mental illness-related TFs.

| Name | Developmental regulation | Mental illness |
| --- | --- | --- |
| Atf1 # | Late up |  |
| Atf3 # | Early down | SZ(+)[^3^](#_ENREF_3), MD(+)[^3^](#_ENREF_3), BP(+)[^3^](#_ENREF_3) |
| Cebpb # | Early down |  |
| Elf1 # | Early up |  |
| Ddit3 | Early down |  |
| Egr2 # | Early down | MD(*)[^4^](#_ENREF_4), BP(*)[^4^](#_ENREF_4) |
| Egr1 # | Early down | SZ(-),MD(-)[^7^](#_ENREF_7) |
| Etv1 | Early down |  |
| Etv5 # | Early down | SZ(-),BP(*)[^8^](#_ENREF_8) |
| Etv6 # | Early up |  |
| Fosl1 | Early down |  |
| Foxg1 # | Early down | SZ[^9^](#_ENREF_9) |
| Id2 # | Early down |  |
| Id4 # | Early down | SZ(+) |
| Irf1 # | Early down |  |
| Jun # | Early down | SZ(+)[^6^](#_ENREF_6),MD(+)[^11^](#_ENREF_11) |
| Klf4 | Early down |  |
| Lhx2 # | Early down |  |
| Elk1 | Late up |  |
| Myc # | Early down | MD[^12^](#_ENREF_12) |
| Nfix # | Early down | BD(*)[^13^](#_ENREF_13) |
| Otx1 | Early down |  |
| Pou3f1 | Early down |  |
| Pou3f3 # | Early down | SZ(+)[^6^](#_ENREF_6) |
| Smad3 | Early down |  |
| Smad2 | Early down |  |
| Sox10 # | Early up | SZ(-),MD(-)[^11^](#_ENREF_11),BP(-)[^14^](#_ENREF_14) |
| Sox6 # | Transient up, Late down | SZ(+)[^6^](#_ENREF_6) |
| Srebf1 | Early up |  |
| Tcf4 # | Transient up, Late down | SZ(*)[^15^](#_ENREF_15),BP(*)[^16^](#_ENREF_16) |

**Supplementary Table S1.** Putative Fez1 promoter-binding TFs in early OL differentiation and dysregulated in psychiatric diseases "-", TFs decreased in patients with mental illness, as compared with healthy control; "+", TFs increased in patients with mental illness, as compared with healthy control; "*", significant SNPs in patients with indicated mental illness; “#” , TFs identified in both OL transcriptome databases. SZ, schizophrenia; MD, major depression; BP, bipolar disorder.

**SUPPLEMENTARY MATERIALS AND METHODS**

**Differentiation of human OL from iPSCs**

Human iPSC colonies were detached with 1 mg/ml collagenase (Invitrogen) treatment for 1 h and suspended in embryoid body (EB) medium, consisting of D-MEM/F12 (Invitrogen), 20% Knockout Serum Replacement (KSR, Invitrogen), 2 mM L-glutamine (Invitrogen), 100 µM MEM NEAA (Invitrogen), 100 µM b-mercaptoethanol (Invitrogen), 2 µM dorsomorphin and 2 µM A-83, in non-treated polystyrene plates for 7 days with a daily medium change. The floating embryoid bodies were transferred to Matrigel (Corning, NY) coated 6-well plates to form neural tube-like rosettes in neural induction medium (NPC medium) consisting of DMEM/F12 (Invitrogen, IL), 1×N2 supplement (Invitrogen), 2 mM L-glutamine (Invitrogen), 100 µM NEAA (Invitrogen), 2 µg/ml heparin (Sigma, St. Louis, MO). On day 23, 1µM purmorphamine (Tocris), 100nM retinoic acid (Sigma) were added to the media for 7 days. On day 30, the rosettes were picked mechanically and transferred to low attachment plates in NPC medium containing 1×B27 supplement (Invitrogen), 10ng/ml bFGF and 1µM purmorphamine. On day 40, suspension culture was switched to oligodendrocyte induction media consisting of DMEM/F12 (Invitrogen), 1×N2 supplement (Invitrogen), 1×B27 supplement (Invitrogen), 60ng/ml T3 (Sigma), 100ng/ml biotin (Sigma), 1µM dibutyryl-cAMP (Sigma), 10ng/ml PDGF-AA (PeproTech, NJ), 10ng/ml IGF-1 (PeproTech), and 10ng/ml NT3 (PeproTech). On day 100, to induce OPC differentiation, progenitor clusters were dissociated with accutase (Invitrogen) at 37°C for 5 minutes and plated onto glass coverslips coated with Matrigel in oligodendrocyte differentiation medium consisting of DMEM/F12 (Invitrogen), 1× N2 supplement (Invitrogen), 1× B27 supplement (Invitrogen), 60ng/ml T3 (Sigma), 100ng/ml biotin (Sigma), 1µM dibutyryl-cAMP (Sigma), 5 ng/ml PDGF-AA (PeproTech), 5 ng/ml IGF-1 (PeproTech), and 5 ng/ml NT3 (PeproTech).

**Morphological analysis**

Primary OLs and CG4 cells were induced for differentiation as indicated in the legends. Light-field and fluorescent images of lentivirus infected primary OLs and fluorescent images of transfected CG4 cells (GFP-positive) were captured from randomly selected microscopic fields using the Olympus IX-51 inverted fluorescent microscope. Processes were categorized into four groups: the primary processes (1°) are directly from the cell body, the secondary processes (2°) are branched from the primary processes, the tertiary processes (3°) are branched from the secondary processes, and the quaternary processes (4°) are branched from the tertiary processes. The morphology of more than 30 randomly selected cells was analyzed from each transfection as described.[^17^](#_ENREF_17)

**Immunoblot analysis and immunofluorescence with antibodies used**

For immunoblot (IB), tissue or cell lysates were prepared and subjected to SDS-PAGE. Band intensity was quantified using Image-Pro Plus software 5.0 (Media Cybernetics). For Immunofluorescence (IF), cryostat slices were prepared from mouse brains. In addition, primary cultured OLs were raised on coverslips and fixed with 4% paraformaldehyde in PBS. For microtubule tracts staining, CG4 cells were fixed with warm fixative containing 3.7% paraformaldehyde (Sigma, St. Louis, MO), 0.05% glutaraldehyde (Grade I, specially purified for use as an electron microscopy fixative, Sigma) and 0.5% Triton X-100 (Thermo Fisher Scientific, Rockford, IL) for 10 min at room temperature.[^18^](#_ENREF_18) Coverslips were blocked with 10% BSA for 30 min and incubated with primary antibodies overnight at 4°C. Secondary antibodies (Fluor 488, 568 and 594 IgG secondary antibodies against rat, rabbit, goat and mouse (1:2000, Thermo Fisher Scientific) were applied for 1 hr at room temperature. Fluorescent images were captured using a Nikon C1 laser-scanning confocal system based on a Nikon inverted microscope (TE300), with a 60× N.A.1.4 Plan Fluor oil-immersion objective. The colocalization of FEZ1 and microtubule tract was analyzed on the same layer from Z-stacks.

Antibodies used for immunoblot and immunofluorescence detections are as follows:

| **Name** | **Company** | **Cat NO.** | **Dilution** | **Method** |
| --- | --- | --- | --- | --- |
| FEZ1 | Novus | [NB100-53816](https://www.novusbio.com/products/fez1-antibody_nb100-53816) | 1:2000 | IB |
| eif5α | Santa Cruz | sc-282 | 1:5000 | IB |
| Flag | Sigma-Aldrich | F1804 | 1:2000 | IB |
| β-actin | Sigma-Aldrich | A5441 | 1:5000 | IB |
| acH3K9 | Millipore | 07-352 | 1:2 000 | IB |
| FEZ1 | Novus | [NB100-53816](https://www.novusbio.com/products/fez1-antibody_nb100-53816) | 1:200 | IF |
| CC1 | Calbiochem | OP80 | 1:100 | IF |
| MBP | Millipore | MAB395 | 1:200 | IF |
| NG2 | Millipore | AB5320 | 1:200 | IF |
| Olig2 | Millipore | AB9610 | 1:500 | IF |
| β-tubulin | Sigma-Aldrich | T5201 | 1:500 | IF |
| 568 Phalloidin | Thermo Fisher Scientific | A12380 | 1:50 | IF |
| Ki67 | Thermo Fisher Scientific | SP6 | 1:500 | IF |

**RT-PCR and Real-Time qRT-PCR.**

Total RNA was isolated using TRIzol (Thermo Fisher Scientific, Rockford, IL). RNA in immunoprecipitated complexes was isolated by phenol-chloroform extraction. Reverse transcription was carried out using Superscript II (Thermo Fisher Scientific, Rockford, IL). Standard RT-PCR was performed to detect FEZ1 mRNA in immunoprecipitated complexes. In addition, RT-qPCR was performed using PerfeCTa SYBR Green Super Mix (Quanta Biosciences, Gaithersburg, MD).  Primers used for RT-PCR and RT-qPCR analysis are as follows:

| **Name** | **Forward 5'-3'** | **Reverse 5'-3'** |
| --- | --- | --- |
| ms Fez1 | GGAAGACCTGCAGATGCTGACAA | TGAGGCTGCTCCAAAGATGAGGT |
| ms Disc1 | GGACTGGCTTATTCGAGAGAAA | TCCTGCTCCTCCAACTCT |
| ms Gapdh | GGTGAAGGTCGGTGTGAAC | CCTTGACTGTGCCGTTGAA |
| Fez1(ChIP) | CTCGCCTTGACCTTGATCTAT | TGGACTGCCCTTTCTATTCTAC |
| rat Sox6 | GGAGATGCGACAGTTCTTCA | GTGTGGTCGTTGCCATAGTA |
| rat Sox10 | TGCTATTCAGGCTCACTACAAGA | ACTGCAGCTCTGTCTTTGGG |
| rat Tcf4 | CACATGAACGTCTGAGCTATCC | GCAGGAAGAGGTGCTGTAAT |
| rat Foxg1 | CAACGGCATCTACGAGTTCA | CACTTGTTGAGGGACAGGTT |
| rat Id2 | AGACCTGGACAGAACCAAACG | CGACATAAGCTCAGAAGGGAAT |
| ms/rat Id4 | GCGATATGAACGACTGCTACA | CAGGATCTCCACTTTGCTGACT |
| rat Fez1 | GGAAGACCTGCAGATGCTGACAA | TGGTGTCATGCAATCCCAGTGATG |
| rat Bdnf | CAGGAGGAATTTCTGAGTGGCCA | GCAGAAGGCCTAAGCAACTTGACA |
| rat P39 | AACCTGGTGTTCGTGTACCTGCT | AGATCTCGTTGCCCATGTAGGAGT |
| rat β-actin | CCTGTATGCCTCTGGTCGTA | CCATCTCTT​GCTCGAAGTCT |

**REFERENCES**

1. Cahoy JD, Emery B, Kaushal A, Foo LC, Zamanian JL, Christopherson KS *et al.* A transcriptome database for astrocytes, neurons, and oligodendrocytes: A new resource for understanding brain development and function. *Journal of Neuroscience* 2008; 28(1): 264-278.

2. Swiss VA, Nguyen T, Dugas J, Ibrahim A, Barres B, Androulakis IP *et al.* Identification of a Gene Regulatory Network Necessary for the Initiation of Oligodendrocyte Differentiation. *Plos One* 2011; 6(4).

3. Weigelt K, Carvalho LA, Drexhage RC, Wijkhuijs A, de Wit H, van Beveren NJM *et al.* TREM-1 and DAP12 expression in monocytes of patients with severe psychiatric disorders. EGR3, ATF3 and PU.1 as important transcription factors. *Brain Behavior and Immunity* 2011; 25(6): 1162-1169.

4. Kim SH, Song JY, Joo EJ, Lee KY, Shin SY, Lee YH *et al.* Genetic association of the EGR2 gene with bipolar disorder in Korea. *Exp Mol Med* 2012; 44(2): 121-129.

5. Perez-Santiago J, Diez-Alarcia R, Callado LF, Zhang JX, Chana G, White CH *et al.* A combined analysis of microarray gene expression studies of the human prefrontal cortex identifies genes implicated in schizophrenia. *J Psychiatr Res* 2012; 46(11): 1464-1474.

6. Narayan S, Tang B, Head SR, Gilmartin TJ, Sutcliffe JG, Dean B *et al.* Molecular profiles of schizophrenia in the CNS at different stages of illness. *Brain research* 2008; 1239: 235-248.

7. Kerman IA, Bernard R, Bonney WE, Jones EG, Schatzberg AE, Myers RM *et al.* Evidence for transcriptional factor dysregulation in the dorsal raphe nucleus of patients with major depressive disorder. *Front Neurosci-Switz* 2012; 6.

8. Williams MJ, Klockars A, Eriksson A, Voisin S, Dnyansagar R, Wiemerslage L *et al.* The Drosophila ETV5 Homologue Ets96B: Molecular Link between Obesity and Bipolar Disorder. *Plos Genet* 2016; 12(6).

9. Won H, de la Torre-Ubieta L, Stein JL, Parikshak NN, Huang J, Opland CK *et al.* Chromosome conformation elucidates regulatory relationships in developing human brain. *Nature* 2016; 538(7626): 523-527.

10. Maycox PR, Kelly F, Taylor A, Bates S, Reid J, Logendra R *et al.* Analysis of gene expression in two large schizophrenia cohorts identifies multiple changes associated with nerve terminal function. *Molecular psychiatry* 2009; 14(12): 1083-1094.

11. Aston C, Jiang L, Sokolov BP. Transcriptional profiling reveals evidence for signaling and oligodendroglial abnormalities in the temporal cortex from patients with major depressive disorder. *Molecular psychiatry* 2005; 10(3): 309-322.

12. Malki K, Koritskaya E, Harris F, Bryson K, Herbster M, Tosto MG. Epigenetic differences in monozygotic twins discordant for major depressive disorder. *Transl Psychiatry* 2016; 6(6): e839.

13. Ikeda M, Takahashi A, Kamatani Y, Okahisa Y, Kunugi H, Mori N *et al.* A genome-wide association study identifies two novel susceptibility loci and trans population polygenicity associated with bipolar disorder. *Mol Psychiatry* 2017.

14. Tkachev D, Mimmack ML, Ryan MM, Wayland M, Freeman T, Jones PB *et al.* Oligodendrocyte dysfunction in schizophrenia and bipolar disorder. *Lancet* 2003; 362(9386): 798-805.

15. Chow TJ, Tee SF, Yong H, Tang PY. Genetic Association of TCF4 and AKT1 Gene Variants with the Age at Onset of Schizophrenia. *Neuropsychobiology* 2016; 73(4): 233-240.

16. Winham SJ, Cuellar-Barboza AB, Oliveros A, McElroy SL, Crow S, Colby C *et al.* Genome-wide association study of bipolar disorder accounting for effect of body mass index identifies a new risk allele in TCF7L2. *Molecular psychiatry* 2014; 19(9): 1010-1016.

17. Chen YT, Tian DH, Ku L, Osterhout DJ, Feng Y. The selective RNA-binding protein quaking I (QKI) is necessary and sufficient for promoting oligodendroglia differentiation. *Journal of Biological Chemistry* 2007; 282(32): 23553-23560.

18. Mabit H, Nakano MY, Prank U, Saam B, Dohner K, Sodeik B *et al.* Intact microtubules support adenovirus and herpes simplex virus infections. *J Virol* 2002; 76(19): 9962-9971.
